# How Much They Know

**Published:** 1892-08

**Authors:** 


### HOW MUCH THEY KNOW.

A recent lecturer on ants and their ways described those of South America, who build immense structures and provide space for the storage of grain. Wood ants, inhabiting hardwood trees, divide their house into forty compartments. Noticing the mining ants, the lecturer said much might be learned from their cleanly habits and their wonderful sanitary arrangements. Some kinds of ants do not keep cows but live entirely on grain. Some facts were given about their interesting harvesting operations—they plant and cultivate a kind of grass called ant rice and are so advanced in civilization that malting is understood by them. Then there are mushroom growing ants who cultivate fungus, and others again who use umbrellas. Several species make raids on the black ants, rob them of their larvæ and compel the poor black ants to be their slaves. In the burying of their dead, ants show wonderful intelligence, having cemeteries and even bury their slaves in a different place from their masters, and are quite up in funeral pageantry. In conclusion the lecturer said that much could be learned from ant life, in their wonderful government, common brotherhood, nursing and care of the young, temperance, and love of fresh air.

W. F. Liesching, writing in the new number of the *Selborne Society's Magazine*, on ants in Ceylon, says he saw one day a string of ants streaming forth, evidently in search of "pastures new." He flicked away the leader and waited to see the result. An immediate halt was made by the foremost ants and a scene of the utmost confusion ensued. The ants from behind kept arriving at the scene of the catastrophe, and there was soon a black crowd of ants huddling and jostling one another. Some detached themselves from the main group and took a turn round, trying to find traces of their leader. At last the tail end of the line arrived, and after brief consultation they all started off again, and a line soon began to unravel itself from the tangled mass, moving back to the hole from which the whole company had so lately started on "pleasure bound or labor all intent."

